# Supplementary material for: Impact of the Sustainable Development Goals on the academic research agenda. A scientometric analysis
Source: PLoS One. 2022 Mar 17;17(3):e0265409. doi: 10.1371/journal.pone.0265409 (PMC8929641; doi:10.1371/journal.pone.0265409)
Supplement: Attachment — Submitted filename: Report_PONE-D-21-28963.pdf [file pone.0265409.s002.pdf]

PONE-D-21-28963 Referee Report

Impact of the Sustainable Development Goals on the academic research agenda. A scientometric analysis

17 December 2021

Summary & Recommendation: This paper offers a scientometric analysis of published articles on the overall theme of sustainable development goals (SDGs) during 2015-2020. To put it in perspective, it is observed that the authors record a total of 5,281 papers contributed by over 22,000 individuals, of which nearly 60% were penned during just two years, 2019-2020. This paper examines, among other, journal concentration, thematic coverage, academic nature of the contributions, and the depth of contribution. From all accounts, it appears to be an exhaustive analysis.

While I make some suggestions for modifications and shortening (see below), I do believe that the paper adds to the knowledge on SDG research around the globe and is publishable in PLOS one. I also have some minor comments, which when addressed are expected to render the contribution sharper.

Major Remarks

(a) *Introduction Section*: While a background on the SDGs (and to a lesser extent, MDGs) is necessary, I find the introduction to the paper with 4 sub-sections far too detailed and delves into far too many dimensions (e.g., merits of some indicators or their inherent amenability to measurement), most of which have little bearings on the analytical methods of the paper. Section 1.4 ('relevance and impact of SDGs on academic research') is however germane to the context of the paper and has a solid claim to retention without much shortening. The prior sections should be drastically reduced to a page each. The present length of nearly 9 pages of introduction is just too much.

(b) *Inclusion Criteria*: The inclusion criteria of an 'article' as relevant to a particular topic of research (e.g., SDGs here) should be laid down first. Often many publications issued by multilateral agencies contain a bunch of data, which becomes very popular with the researchers, but NOT for any analysis that the document may contain, merely the dataset. Either these should be left out of a scientometric analysis or included in a review dedicated only to databases on a theme. Here the author states that many top-cited papers included documents sponsored by UN Agencies or the World Bank (WB). The concern here is that some or perhaps most of these would be referenced mainly on account of data and related information.

(c) *Sample Properties*: A major observation is that of the 5K-plus articles, nearly 71% of the contributions (Table 1) focussed on 'the environment' broadly interpreted which essentially covers only 4 (Goals #3, 6, 7 and 13) out of 17 SDGs. Thus, a majority of

the articles analysed in the paper have little to do with a majority of the sustainable goals (namely poverty elimination, hunger, quality education, animal kingdom, or justice and institutions. It would therefore seem that somehow the research to date has been lopsided in its focus. Some discussion is necessary if this claim is actually true of the universe of all published research on the totality of SDGs. Or we are dealing with a biased sample.

(d) *Top Journals*: Table 4 enumerates the top 18 journals that has published most articles on SDGs. However, in the discussion part (section 4), we discover that only 6 articles received over 100 citations each. Of this group, 3 were published in *Nature*, but one each in *Lancet*, *Science,* & *Sustainable Development*. To the extent citations may be used to reflect the standing of a journal, *Nature* appears to trump all others, even though Lancet is ranked the highest in terms of the 'impact factor'. *Nature* does not even figure in the list of top 18 journals in Table 4. These observations should be flagged in the context of the discussion based on Table 4.

Minor Remarks

(i) I find the 'Discussion' section to be on lengthy side, with 5 pages devoted to it. Perhaps 3 to 3.5 would have been adequate to convey the main points to the reader.

(ii) I detected a few, not many, typos, which shall be take care of while 'copy-editing'.
