# Supplementary material for: Impact of the Sustainable Development Goals on the academic research agenda. A scientometric analysis
Source: PLoS One. 2022 Mar 17;17(3):e0265409. doi: 10.1371/journal.pone.0265409 (PMC8929641; doi:10.1371/journal.pone.0265409)
Supplement: Attachment — Submitted filename: Response to Reviewers.doc [file pone.0265409.s003.doc]

We sincerely appreciate the reviewers’ suggestions and comments for the previous version of this manuscript. We have studied them thoroughly and have revised the manuscript accordingly. This report summarizes our responses to all the comments offered by the reviewers.

**REVIEWER 1**

| **Suggestions/comments from the Reviewer** | **Response from the Author(s)** |
| --- | --- |
| Really I want to give thanks for your great work and really it’s a great methodology and nice ideas for the analysis and looking forward for more ideas and researches relate to the same related topics. | Thank you so much for your support and for highlighting our scientific approach to provide guidance to future researchers in the field. Your comments are really encouraging. |

**REVIEWER 2**

| **Suggestions/comments from the Reviewer** | **Response from the Author(s)** |
| --- | --- |
| This paper offers a scientometric analysis of published articles on the overall theme of Sustainable Development Goals (SDGs) during 2015-2020. To put it in perspective, it is observed that the authors record a total of 5,281 papers contributed by over 22,000 individuals, of which nearly 60% were penned during just two years, 2019-2020. This paper examines, among other, journal concentration, thematic coverage, academic nature of the contributions, and the depth of contribution. From all accounts, it appears to be an exhaustive analysis. | Thank you so much for highlighting our scientific approach and our contributions to provide guidance to future researchers in the field. Your comments are really encouraging. |
| While I make some suggestions for modifications and shortening (see below), I do believe that the paper adds to the knowledge on SDG research around the globe and is publishable in PLOS one. | Thank you so much for your support towards the publication of our contribution. |
| I also have some minor comments, which when addressed are expected to render the contribution sharper.  Comments and suggestions are included in the referee report attached herewith, which can be sent to the authors in entirety | Again, we want to thank you for taking the time to suggest improvements to our manuscript. In the following points, we will address all of your comments and suggestions one by one. |
| (a) Introduction Section: While a background on the SDGs (and to a lesser extent, MDGs) is necessary, I find the introduction to the paper with 4 sub-sections far too detailed and delves into far too many dimensions (e.g., merits of some indicators or their inherent amenability to measurement), most of which have little bearings on the analytical methods of the paper. Section 1.4 (‘relevance and impact of SDGs on academic research’) is however germane to the context of the paper and has a solid claim to retention without much shortening. The prior sections should be drastically reduced to a page each. The present length of nearly 9 pages of introduction is just too much. | We want to appreciate your support to our background analysis, while also recognizing its need to be shortened. We have proceeded to deeply simplify this whole section, keeping their contributes while making it softener for readers.  First, and following your wise suggestion, we have melted previous Section 1.1. and Section 1.2. into one single section, now renamed: “Section 1.1. From the Millennium Agenda to the 2030 Agenda and the Sustainable Development Goals (SDGs)”.  We have also cut its extension almost by half, as suggested by you. You can see such changes in lines 43-119.  Section 1.3, now Section 1.2. on “Towards a categorization of the SDGs”, has also been shortened following your wise suggestion. Now, it contributes to a softer reading of the paper, while still provides the categorization that is followed in the discussion section.  We really appreciate your strong support of Section 1.4., which we have also shortened lightly.  Overall, this section has passed from 9 pages to less than 7 pages, which undoubtedly has made it clearer and easier for readers. If more reduction is needed, please feel free to provide further suggestions. |
| (b) Inclusion Criteria: The inclusion criteria of an ‘article’ as relevant to a particular topic of research (e.g., SDGs here) should be laid down first. Often many publications issued by multilateral agencies contain a bunch of data, which becomes very popular with the researchers, but NOT for any analysis that the document may contain, merely the dataset. Either these should be left out of a scientometric analysis or included in a review dedicated only to databases on a theme. Here the author states that many top-cited papers included documents sponsored by UN Agencies or the World Bank (WB). The concern here is that some or perhaps most of these would be referenced mainly on account of data and related information. | Thank you for pointing this issue out, which offers us the possibility to better explain the retrieval of our database and the nature of the contributions included on it.  Indeed, in our database, there are no datasets or other publications provided by multilateral agencies. Our database only contains contributions published in the Social Citation Index-Expanded (SCI-E) and the Social Sciences Citation Index (SSCI). Thus, only contributions (articles, reviews, editorials, etc.) directly written by researchers in the field.  When some documents published by UN Agencies or the World Bank (WB) appear in our analyses, is always in the case of citation and co-citation analyses. It is, when looking not at the contributions included in our database, but when looking at the documents cited in such contributions. In fact, it is one of the main findings of this research process: that academic researchers often rely more on documents of normative nature (reports by UN agencies, etc.) that on peer-review documents when providing the background of their empirical research.  Anyway, following your accurate observation, we have further clarified the details and criteria of our information retrieval. You can find it in the methodology section, lines 246-254:  “In methodological terms, this research applies scientometrics as a meta-analytical means to study the evolution of documented scientific knowledge on the Sustainable Development Goals (76–81), taking as a secondary source of information academic contributions (i.e. articles, reviews, editorials, etc.) indexed in the Web of Science (WoS). To ensure that only peer-reviewed contributions authored by individual researchers are retrieved and that such publications have a worldwide prestige assessment, all of them should be published on journals indexed in the Journal Citation Report (JCR), either as part of the Sciences Citation Index Expanded or the Social Sciences Citation Index (82–84)”. |
| (c) Sample Properties: A major observation is that of the 5K-plus articles, nearly 71% of the contributions (Table 1) focussed on ‘the environment’ broadly interpreted which essentially covers only 4 (Goals #3, 6, 7 and 13) out of 17 SDGs. Thus, a majority of the articles analysed in the paper have little to do with a majority of the sustainable goals (namely poverty elimination, hunger, quality education, animal kingdom, or justice and institutions. It would therefore seem that somehow the research to date has been lopsided in its focus. Some discussion is necessary if this claim is actually true of the universe of all published research on the totality of SDGs. Or we are dealing with a biased sample. | Thank you for this observation. Indeed, the strong focus of academic researchers on the environmental aspects of the SDGs is one of the main findings of this study. Certainly, there is no bias in the selection of the sample, which was carefully scrutinized by all authors.  In our opinion, this focus is well documented in different parts of the manuscript, but after re-reading, we have also included it explicitly in the first analysis provided in the discussion section, to reinforce this finding. You can find it in lines 460-464:  “Thus, the size of the body of literature and the broad spectrum of topics more than covers the four perspectives of analysis that are relevant in research on the SDGs, according to Hajer et al. (19): planetary boundaries, the safe and just operating space, the energetic society and, last, green competition. However, results also highlight a strong focus on the environmental aspects of the SDGs, which undoubtedly concentrate the most contributions”. |
| (d) Top Journals: Table 4 enumerates the top 18 journals that has published most articles on SDGs. However, in the discussion part (section 4), we discover that only 6 articles received over 100 citations each. Of this group, 3 were published in Nature, but one each in Lancet, Science, & Sustainable Development. To the extent citations may be used to reflect the standing of a journal, Nature appears to trump all others, even though Lancet is ranked the highest in terms of the ‘impact factor’. Nature does not even figure in the list of top 18 journals in Table 4. These observations should be flagged in the context of the discussion based on Table 4. | Thank you for pointing out this situation, which clearly shows the detailed revision you have provided. There is a reason for Nature not appearing in Table 4, but it was certainly not included in the manuscript.  Table 4 takes the top 18 journals as they are the ones included in the Bradford’s nucleus. It is, these are the journals that published more papers. Once these journals are selected, they are ranked by combining the number of contributions and the influence they have had in the literature. It is, we generate our “Prominence ranking” by combining two elements: number of papers and their reach (using citations as a proxy of it).  If Nature would have been included in the Bradford’s nucleus, given the mean citation of the contributions published in this journal, it would have been a top 5 journal in our Prominence Ranking.  To better reflect this information and what happens with Nature, we include:   - In lines 329-331: “As shown in Table 4, we have developed a “Prominence ranking” by weighting article production by impact factor., This metric shows The Lancet, with only 40 articles on SDGs, as the most relevant journal […]”. - In lines 423-425: “It is worth noting that some of these top journals may not be listed in Table 4 as they are not included in the Bradford’s nucleus, due to their comparatively low number of contributions published”. |
| (i) I find the ‘Discussion’ section to be on lengthy side, with 5 pages devoted to it. Perhaps 3 to 3.5 would have been adequate to convey the main points to the reader. | Once again, after review, we agree that some aspects of the Discussion section could well be reduced. We have done so in the following parts of this section:   - Lines 451-455. - Lines 480-483. - Lines 510-512. - Lines 522-527. |
| (ii) I detected a few, not many, typos, which shall be take care of while ‘copy-editing’. | Thank you for this observation. We have re-send our contribution to AJE, so they could revise once again their translation and such typos. They have included them without track changes, but we have also revised the manuscript and we have not found any significant typo by our own. Of course, may you detect any further typos, we will be willing to take a care look at them |
|  | We want to thank Reviewer 2 for the time dedicated to review our contribution. Your comments and suggestions have undoubtedly contributed to the improvement of our study. |
